# Evaluation of glycerol encapsulated with alginate and alginate-chitosan polymers in gut environment and its resistance to rumen microbial degradation

**DOI:** 10.5713/ajas.18.0110

**Published:** 2018-07-26

**Authors:** Ramadan Gawad, Vivek Fellner

**Affiliations:** 1 Department of Animal Science and Interdepartmental Nutrition Program, North Carolina State University, Raleigh, NC 27695-7621, USA; 2 Food Technology and Nutrition Division, National Research Center, Dokki, Giza 12622, Egypt

**Keywords:** Glycerol, Alginate, Chitosan, Encapsulation, Rumen, pH

## Abstract

**Objective:**

To determine the effect of gut pH and rumen microbial fermentation on glycerol encapsulated in alginate and alginate-chitosan polymers.

**Methods:**

Glycerol was encapsulated at 2.5%, 5%, 7.5%, or 10% (w/w) with sodium alginate (A) and alginate-chitosan (AC) polymers. Surface morphology and chemical modifications of the beads were evaluated using scanning electron microscopy and Fourier transform infrared (FTIR) spectra. Encapsulation efficiency was determined at the 5% glycerol inclusion level in two experiments. In experiment 1, 0.5 g of alginate-glycerol (AG) and alginate-chitosan glycerol (ACG) beads were incubated for 2 h at 39°C in pH 2 buffer followed by 24 h in pH 8 buffer to simulate gastric and intestinal conditions, respectively. In experiment 2, 0.5 g of AG and ACG beads were incubated in pH 6 buffer at 39°C for 8 h to simulate rumen conditions. All incubations were replicated four times. Free glycerol content was determined using a spectrophotometer and used to assess loading capacity and encapsulation efficiency. An *in vitro* experiment with mixed cultures of rumen microbes was conducted to determine effect of encapsulation on microbial fermentation. Data were analyzed according to a complete block design using the MIXED procedure of SAS (SAS Institute, Cary, NC, USA).

**Results:**

For AG and ACG, loading capacity and efficiency were 64.7%, 74.7%, 70.3%, and 78.1%, respectively. Based on the FTIR spectra and scanning electron microscopy, ACG treatment demonstrated more intense and stronger ionic bonds. At pH 6, 36.1% and 29.7% of glycerol was released from AG and ACG, respectively. At pH 2 minimal glycerol was released but pH 8 resulted in 95.7% and 93.9% of glycerol released from AG and ACG, respectively. *In vitro* microbial data show reduced (p<0.05) fermentation of encapsulated glycerol after 24 h of incubation.

**Conclusion:**

The AC polymer provided greater protection in acidic pH with a gradual release of intact glycerol when exposed to an alkaline pH.

## INTRODUCTION

Glycerol is a major byproduct of biodiesel [1]. The increased demand for biodiesel as an alternative energy source has created a large surplus of glycerol [2]. Traditional uses of glycerol are saturated limiting the production of biodiesel as a sustainable source of energy [3]. Glycerol has a high energy content and is recognized as a safe feed ingredient [4]. Low price and surplus quantity makes glycerol economically competitive with other grains as a feed supplement for livestock [5]. However, the amount of glycerol that can be included in the diet is limited because it is rapidly fermented in the rumen which can have a negative effect on microbial fermentation [6,7]. Encapsulation can reduce the amount of glycerol made available in the rumen thereby minimize the negative impact on fermentation and allow greater amounts to be included in livestock feeds. Protecting dietary supplements from ruminal environment has been a common approach to allow greater inclusion of supplements and increase nutritional value of the ingredient [8,9]. Encapsulating glycerol to bypass rumen fermentation has not previously been investigated.

Our objective was to evaluate alginate and alginate-chitosan as encapsulating polymers for glycerol. Alginate and chitosan are biocompatible, non-toxic and have selective biodegradability [10]. Chitosan is less soluble in alkaline pH and alginate is insoluble at low pH. We hypothesized that compared to alginate alone the alginate-chitosan mix would result in a stronger matrix and be more effective as an encapsulating polymer.

## MATERIALS AND METHODS

### Materials

Medium viscosity (≥2,000 cp) sodium alginate isolated from brown algae, with a molecular weight between 80 and 120 kDa and a mannuronic to guluronic acid ratio of 1.56 was purchased from Sigma-Aldrich, St. Louis, MO, USA. The polymer chitosan (molecular weight: 190 to 310 kDa, deacetylation degree 75% to 85%, and viscosity of 200 to 800 cp) was also purchased from Sigma-Aldrich, USA. High purity grade glycerol (99.8%) and calcium chloride, as a cross linking agent, were purchased from Fisher Scientific, Pittsburgh, PA, USA. All other chemical reagents were of analytical grade.

### Preparation of beads

Glycerol was added at 2.5%, 5%, 7.5%, or 10% to each of two bead solutions. One bead solution contained 2.5% (w/v) sodium alginate in distilled water. The other bead solution contained a chitosan solution (1.25% w/v) in dilute acetic acid (1%) mixed with sodium alginate (2.5% w/v). The alginate-glycerol (AG) and alginate-chitosan glycerol (ACG) beads were prepared by adding the different levels of glycerol into a 500 mL wide mouth beaker containing the two bead solutions, respectively. The bead solution mixture was allowed to stir at 500 rpm for an hour, then transferred into a separating funnel and dropped through an 18-gauge needle (38 mm length, 1.27 mm diameter) from a 100 mL glass syringe into a wide mouth beaker containing a 3% (w/v) calcium chloride solution. The beads were allowed to mix under gentle stirring (10 rpm) for an hour to harden. After one hour, the beads were filtered through a cheesecloth and rinsed under distilled water. Following adequate rinsing, the beads were air dried at 23°C for 12 hours and stored in air tight containers for subsequent experiments.

### Effect of glycerol concentration on loading capacity and encapsulation efficiency

Representative samples of AG and ACG beads at each level of glycerol inclusion i.e. 2.5%, 5%, 7.5%, or 10% were prepared, in triplicate, to determine free glycerol content according to the method described by Bondioli and Della Bella [11] at 412 nm using a UV spectrophotometer (Shimadzu UV1601, Tokyo, Japan). A calibration curve (R^2^ = 0.996) was generated from glycerol standard solutions at known concentrations. The loading capacity (LC) and encapsulation efficiency (EE) of the AG and ACG glycerol beads were calculated based on the equations below:

$$\begin{array}{l} \text{LC }(\%)=(\text{glycerol content in beads}/\text{weight of beads})\times 100\\ \text{EE }(\%)=(\text{glycerol content in beads}/\text{initial amount of glycerol added})\times 100 \end{array}$$

### Scanning electron microscopy analysis

The surface morphology of the beads was observed using a field emission scanning electron microscopy (SEM) (FE-SEM, JEOL, 6400F, Japan). The beads were mounted on an appropriate stub, coated with carbon and gold, and photographs were taken at a magnification of 50×, 1,000×, and 10,000×. The working distance was maintained at 20 mm and the acceleration voltage was 5 kV.

### Structural analysis by Fourier transform infrared spectroscopy

Fourier transform infrared (FTIR) spectrum of glycerol, alginate, chitosan, AG, and ACG beads were recorded using a transmittance mode iS50 Thermo Nicolet Nexus 670 FTIR (Thermo Scientific, Waltham, MA, USA) with a built-in diamond crystal. The absorbance pattern of diamond typically seen between 1,900 and 2,400 cm^−1^ was erased. No bonds were observed in that area. Analysis was performed within the spectral region of 400 to 4,000 cm^−1^ with 32 scans recorded at 4 cm^−1^ resolution. FTIR spectra from the different bead preparations were compared to evaluate chemical modifications.

### Glycerol release *in-vitro*

Glycerol release experiments were performed at pH 6, 2, and 8 to simulate rumen, gastric, and intestinal conditions, respectively. The gastric buffer (pH 2) was prepared by mixing 50 mL of a 0.2 M KCl with 13 mL of 0.2 M HCl and brought to a total volume of 200 mL with distilled water. The intestinal buffer (pH 8) was prepared by mixing 100 mL of a 0.1 M KH_2_PO_4_ with 93.4 mL of 0.1 M NaOH and brought to a total volume of 200 mL with distilled water and the rumen buffer (pH 6) was prepared by mixing 100 mL of 0.1 M KH_2_PO_4_ with 11.2 mL of 0.1 M NaOH and brought to a total volume of 200 mL with distilled water.

The ability of the encapsulating polymers to protect glycerol from acidic and alkaline hydrolysis was assessed in two separate *in vitro* experiments. In experiment 1, acidic and alkaline phases were studied sequentially by placing 0.5 g samples of AG and ACG beads inside conical flasks (200 mL), containing 100 mL of pH 2 buffer. Four flasks were prepared per treatment to provide for additional replications. All flasks were incubated at 39°C for 2 h to simulate gastric digestion. After 2 h, a sample was taken and stored for subsequent glycerol analysis. The beads were rinsed with distilled water and transferred to pH 8 buffer at 39°C and incubated until 24 h to simulate intestinal conditions. In experiment 2, 0.5 g samples of AG and ACG beads were incubated in 200 mL of pH 6 buffer at 39°C for 8 h to simulate rumen conditions. Each treatment was repeated four times to provide for additional replications. Samples were withdrawn at 2 h intervals and stored for subsequent glycerol analysis. Free glycerol content was determined at 412 nm using a spectrophotometer [11].

### *In vitro* fermentation using mixed rumen microbes

*In vitro* fermentation was conducted using batch cultures of mixed rumen microorganisms. Diets consisted of pelleted alfalfa and corn (60:40). Free (unprotected) glycerol and encapsulated glycerol beads were included to provide glycerol at 25% of dry matter. Glycerol, AG and ACG were substituted for corn. Treatments included: i) Con (no glycerol), ii) G (free unprotected glycerol), iii) AG (alginate beads), and iv) ACG (alginate-chitosan beads). Donor animal for rumen contents was a non-lactating cannulated Holstein cow fed a predominantly forage diet. Rumen contents were collected 2 h after the morning feeding and immediately transported to the laboratory in a thermal flask. In the lab, rumen contents were strained through four layers of cheesecloth and mixed with a buffer solution [12] in a 1:2 ratio (vol/vol). The rumen contents and buffer were maintained at 39°C and CO_2_ was purged through the flasks at all times to minimize exposure to O_2_. Individual treatment substrates were quantitatively weighed into 120 mL bottles, in triplicate, for each time period and allowed to ferment for 0 and 24 hours. Bottles containing only the rumen inoculum and no substrate served as blanks. Forty mL of the buffered rumen inoculum were added into each bottle under constant flow of CO_2_. Bottles were immediately sealed with rubber lined screw caps and placed in a 39°C water bath. The 0 h samples were sealed and immediately placed on ice to stop fermentation. After 24 h, samples were removed from the water bath, placed on ice and culture pH was recorded prior to storing bottles in a walk in freezer at −20°C for further chemical analysis. Short chain fatty acids (SCFAs) were quantified using a gas chromatograph (Model 3380, Varian, Walnut Creek, CA, USA) using a fused silica capillary column (Nukol; Supelco Inc., Bellefonte, PA, USA). The amount of SCFA in each batch culture after 24 h of incubation was corrected for the amount of SCFA in the rumen fluid used as inoculum.

### Statistical analysis

Data from the encapsulation experiment were analyzed according to a complete block design using the MIXED procedure of SAS (SAS Institute, Cary, NC, USA). The statistical model included fixed effect of polymers, amount of glycerol and the polymer×glycerol interaction. Replicate was included as the random term. Data from the glycerol release experiments were analyzed according to a complete block design using the MIXED procedure of SAS. The statistical model included the fixed effects of polymer, pH and incubation time and the respective interactions. Data from the *in vitro* fermentation with mixed rumen microbes was analyzed by hour (0 vs 24) as a complete block design. The MIXED procedure of SAS was used to separate means within the hour. The statistical model included the fixed effect of treatment. Replicate was included as random effect. Differences among treatment means in all experiments were determined by the LSmeans/pdiff option. Differences were considered significant at p<0.05 with trends defined as 0.05<p<0.10.

## RESULTS

### Formation and physical examination of beads

**Figure 1:**
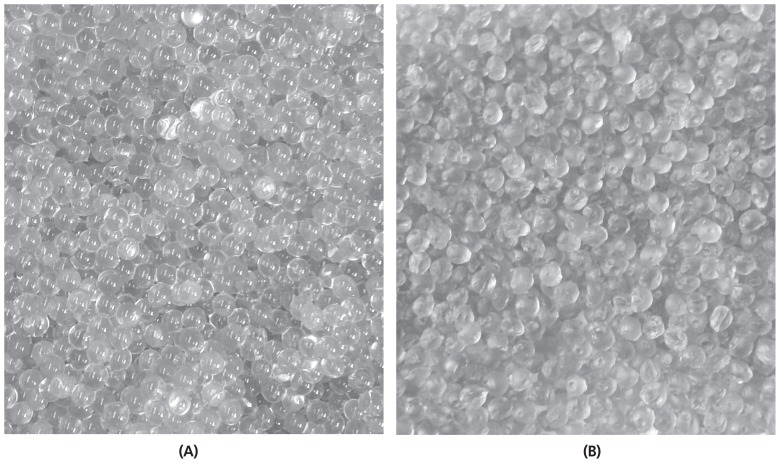
Glycerol beads (*X̄* diameter = 3 mm) encapsulated in alginate (AG). Beads were prepared by dripping 5% (w/v) glycerol and 2.5% (w/v) sodium alginate into 3% (w/v) CaCl_2_ solutions. Figure shows photographs of samples taken fresh immediately after filtering through cheesecloth and rinsed in water (A) and samples after drying at 23°C for 12 h (B).

Images of wet and dry beads are presented in Figure 1. Fresh beads (Figure 1A) were round in shape with a smooth and homogeneous surface. The drying process collapsed the beads and reduced their dimensions (Figure 1B). The loss of water made the beads slightly darker in color and more ellipsoidal with a rougher surface. Both, the AG and ACG beads were similar in color and size. The ionotropic gelation method used in this study to encapsulate glycerol through an 18-gauge needle resulted in fresh bead size of approximately 3 mm in diameter with a spherical appearance and a smooth outer matrix (Figure 1).

### Glycerol loading capacity and encapsulation efficiency

**Figure 2:**
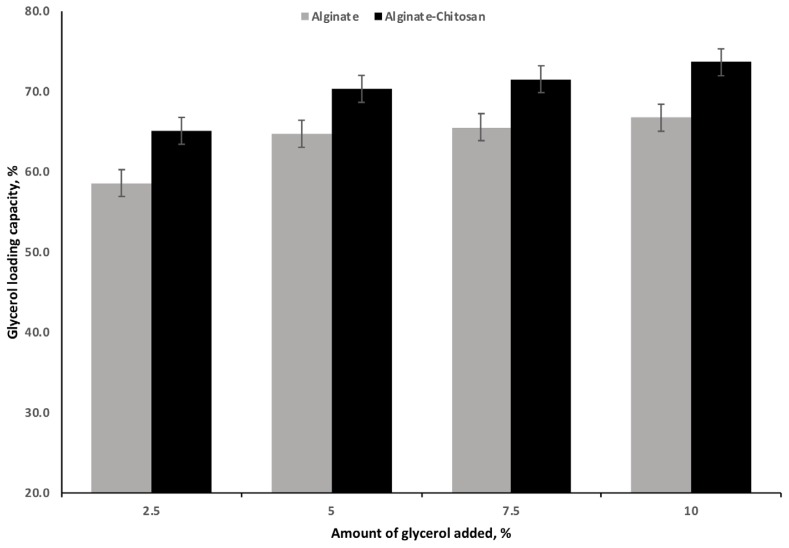
Effect of increasing levels of glycerol on loading capacity of glycerol encapsulated with 2.5% (w/v) alginate or 2.5% (w/v) alginate and 1.25% (w/v) chitosan mixture as encapsulating polymers (n = 3). Effect of polymer (p<0.001). Effect of glycerol (p<0.001).

There was a significant effect of the concentration of glycerol and polymer treatment on glycerol LC (Figure 2). Increasing the concentration of glycerol increased (p<0.001) the amount of glycerol loaded irrespective of the polymer treatment. The greatest increase in LC, irrespective of polymer, occurred when the level of glycerol was raised from 2.5% to 5%. Further increases in the level of glycerol concentrations (7.5% and 10%) did not have an appreciable increase in glycerol LC for either AG or ACG treatments. Compared with AG, the ACG treatment significantly increased LC at all glycerol concentrations (Figure 2). The maximum LC was 73.6% and 66.7% for ACG and AG beads, respectively, when glycerol was included at 10%.

**Figure 3:**
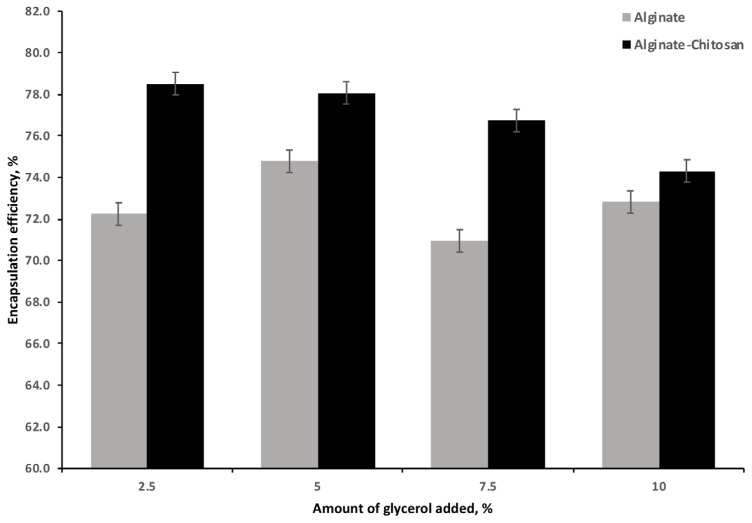
Effect of increasing amounts of glycerol on efficiency of encapsulation by alginate or alginate chitosan polymers (n = 3). Effect of polymer (p<0.001). Effect of glycerol (p<0.001). Effect of polymer · glycerol (p<0.001).

The EE for the AG and ACG beads is illustrated in Figure 3. The ACG beads resulted in the highest efficiency (78.5%) compared to the AG beads (72.2%) at the 2.5% of glycerol addition. Increasing the glycerol LC resulted in a significant and linear decrease in EE with the ACG treatment; the effect of AG was variable but significantly lower than the ACG at all glycerol levels.

### Scanning electron microscope characterization

**Figure 4:**
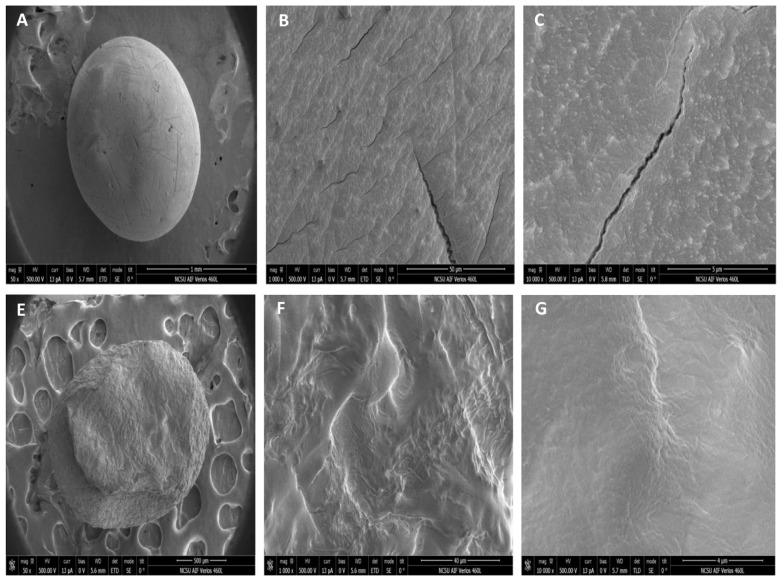
Scanning electron microscopy photographs of glycerol beads encapsulated in alginate (AG) or alginate-chitosan (ACG) polymers. Surface morphology of the bead preparations is depicted at scanning intensity of 50×, 1,000×, and 10,000× for AG (A, B, C) and ACG (E, F, G), respectively.

SEM of AG (A, B, C) and ACG (E, F, G) beads are illustrated in Figure 4. To better depict the molecular structure beads were scanned at three intensities (50×, 1,000×, and 10,000×) as illustrated in A, B, C for the AG beads and E, F, G for the ACG beads. At lower magnification the AG beads (Figure 4A) were smoother and more spherical compared to ACG beads (Figure 4E) that had a more rough and undulating surface. At higher magnification however, we can clearly observe the presence of cracks present in the AG beads (Figure 4C) but not in ACG beads (Figure 4G).

### Fourier transform infrared analysis

**Figure 5:**
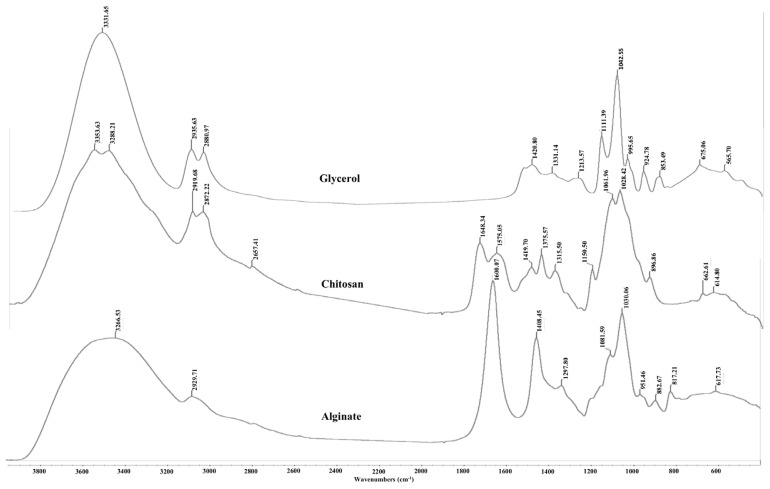
Fourier transform infrared spectra of free glycerol, chitosan and sodium alginate. Peaks characteristic of OH bonding, CH and CO stretching appear at ~3,300 cm^−1^, 2,900 cm^−1^, and 1,100 cm^−1^, respectively and were present in all three compounds. A strong band at 1,648 cm^−1^ characteristic of the amide vibration was recorded in chitosan.

**Figure 6:**
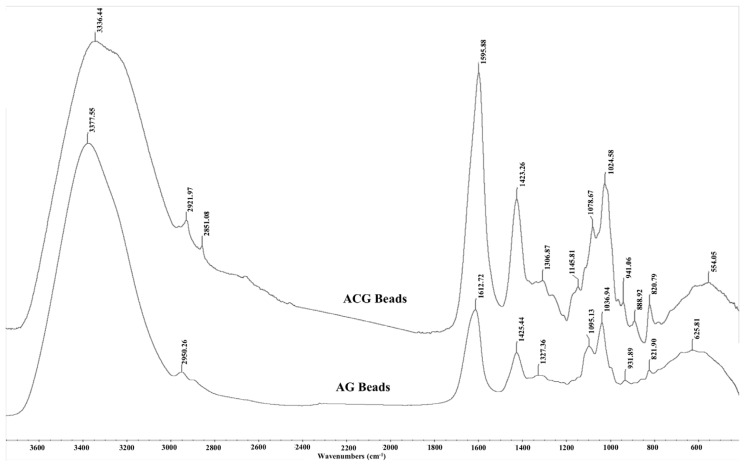
Fourier transform infrared spectra of glycerol encapsulated in alginate (AG) or alginate-chitosan (ACG) polymers. Spectral position of peaks are relatively similar to parent compounds i.e. glycerol, chitosan, and alginate. Greater intensity and narrower peaks in ACG reflect stronger ionic bonds between amine groups in chitosan and carboxylate groups of alginate.

The FTIR spectra of glycerol, alginate, chitosan powder and AG and ACG beads are shown in Figures 5 and 6, respectively. The large peak in glycerol at 3,331.65 cm^−1^ is due to the characteristic OH stretching modes. The two peaks at 2,935 and 2,880 cm^−1^ are reflective of the CH stretching vibrations of glycerol whereas the peaks between 1,500 and 1,200 cm^−1^ are due to the CH bending. The C-O stretching is shown in peaks between 1,200 and 900 cm^−1^. Alginate powder spectrum also shows a broad band at 3,266 cm^−1^ related to OH stretching with strong intra and/or inter hydrogen bonding. The peak at 2,929 cm^−1^ is ascribed to the overlapping symmetrical and asymmetrical C-H stretching vibration of aliphatic chains (-CH_2_-, -CH_3_). The asymmetric and symmetric vibrational modes of carboxylate ions (COO^−^) were recorded at 1,600 and 1,408 cm^−1^, respectively. The vibrational mode at 1,081 cm^−1^ was attributable to the C–O stretching vibration of pyranosyl ring. Due to its polysaccharide structure the (C-O-C) stretching vibration of sodium alginate was manifested at 1,030 cm^−1^. The spectrum of chitosan is characterized by broad and intense dimeric bands at 3,353 to 3,288 cm^−1^ (hydrogen bonded OH stretching overlapped with N-H stretching bands). Chitosan also showed bands at 2,919 and 2,872 cm^−1^ due to asymmetric stretching of -CH_2_- and -CH_3_ groups. The N-H deformation band of chitosan was found at 1,559 cm^−1^. The strong bands at 1,648 and 1,575 cm^−1^ are ascribed to the amide I (C=O stretching) and amide II (N-H bending modes) and C-N stretching vibrations of chitosan [13,14]. A characteristic peak at 898 cm^−1^ was observed due to the β-1–4 glycosidic linkage bond in chitosan.

### Release experiments

**Figure 7:**
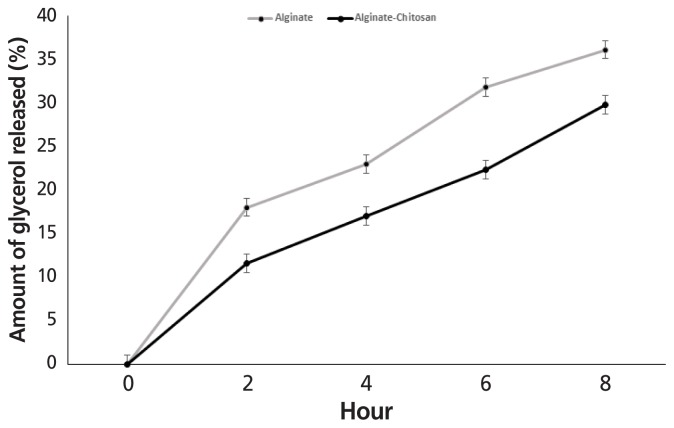
Effect of incubating glycerol encapsulated in alginate (AG) or alginate-chitosan (ACG) polymers in a buffer with a pH of 6.0 to simulate glycerol release in the rumen (n = 3).

Glycerol release from AG and ACG beads when incubated in rumen buffer at pH 6 and 39°C for 8 h is shown in Figure 7. Irrespective of polymer treatment, exposing beads to rumen pH for 8 h resulted in a steady release of glycerol. However, the amount of glycerol that escaped from the AG beads was significantly greater when compared to ACG. At end of 8 h, 36.1% of glycerol was released from the AG beads compared with 29.8% for the ACG beads.

**Figure 8:**
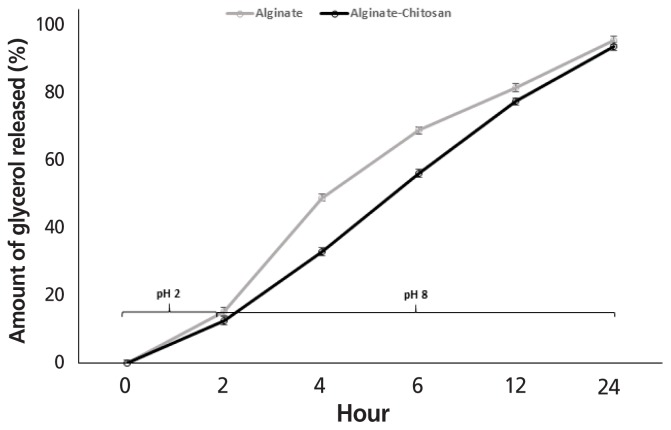
Effect of incubating glycerol encapsulated in alginate (AG) or alginate-chitosan (ACG) polymers in a buffer with a pH of 2 and 8 to simulate glycerol release in the gastric and intestine, respectively (n = 3).

Glycerol release from AG and ACG beads when incubated at 39°C in gastric buffer at pH 2 for 2 h and then in intestinal buffer at pH 8 until 24 h is shown in Figure 8. At the end of 2 h, the amount of glycerol released in gastric buffer tended (p<0.08) to be similar between AG and ACG beads and averaged 15.4% and 12.6%, respectively. Following gastric conditions, both beads were exposed to intestinal buffer for an additional 22 h. The period from 2 to 6 h simulated retention time in the small intestine and 12 to 24 h simulated retention in the large intestine including colon and cecum of herbivores. During the initial 2 to 6 h corresponding to the small intestinal retention time, 69% of the glycerol was released (p<0.05) from AG compared to 56% from the ACG beads. However, by 24 h, more than 90% of the glycerol was released irrespective of the polymer treatment (p>0.10). At the higher pH of the intestinal buffer simulating the small and large intestines substantial amounts of glycerol escaped from both the AG and ACG beads and polymer treatment did not have an effect (p>0.10). More than 90% of the glycerol was released from both AG and ACG beads at the end of 24 h of incubation in the intestinal buffer. During the initial 6 h of incubation glycerol release seemed more rapid from the AG beads compared to the ACG beads. This suggests that ACG would result in a gradual supply of glycerol within the small intestine compared to a rapid release rat with the AG beads.

### *In vitro* fermentation with mixed rumen microbes

There was no difference (p>0.10) in culture pH across all treatments at 0 h (Table 1). As expected, pH dropped at 24 h and was similar for all treatments. After 24 h of incubation, total SCFA increased (p<0.05) in Con and G. Acetate was significantly reduced in cultures receiving G (free glycerol) when compared with either the Con or AG and ACG treatments. In contrast, molar proportion of propionate were much higher (p<0.05) in cultures receiving G compared to either Con or AG and ACG. The lower acetate and higher propionate resulted in a significantly lower acetate:propionate ratio in cultures receiving free glycerol (G). Visual observations indicated that the beads behaved similar to the particulate fraction and stayed in suspension during the initial stages of fermentation. At the end of 24 h of incubation some beads remained in suspension while some sank to the bottom with the more dense material. In this study, we did not measure the density of the beads. However, based on the observed behavior of the beads in rumen cultures, we can speculate that the specific gravity of the beads was greater than 1 and less than 1.5. Beads used in this study were on the average 3 mm in diameter. Particles 3 mm in size with specific gravities ranging between 1 and 1.3 are common within the rumen environment [15].

## DISCUSSION

Among several factors, gauge of the needle and concentration of CaCl_2_, can alter the size, shape and chemical characteristics of beads [16]. Size of beads ultimately is dictated by their intended application. We used an 18-gauge needle to prepare beads with uniformity and that would hold the maximum amount of glycerol yet be reasonably small in size to mix adequately when included in practical livestock diets. The viscosity of glycerol precludes the use of smaller gauge needles without some form of mechanical pressure. The size and appearance of AG and ACG beads were comparable to results reported by other investigators who used similar polymers for encapsulation [16,17]. The irregularity and rough surface in alginate-chitosan beads is explained by the adhesive properties of chitosan, more rapid ionic interaction and increased polyanion-polycation complex aggregates [18–20].

Factors that affect EE and LC include polymer concentration, crosslinking agent concentration and cross linking time [16]. Studies have reported variable values for LC and EE with alginate alone or in combination with other polymers. Soliman et al [21] used alginate to encapsulate different kinds of essential oils and reported that the alginate microspheres under optimized conditions provided LC of 22% to 24% and EE of 90% to 94% for different types of essential oils. Spherical microspheres of theophylline using sodium alginate as the hydrophilic carrier were reported to have entrapment efficiency of 70% to 93% [22]. Encapsulation of liquid smoke flavoring with calcium alginate and calcium alginate–chitosan resulted in encapsulation capacities above 96% [23]. Encapsulation efficiencies for linseed oil and other plant oils with calcium alginate were 88% and 71% to 75%, respectively [24]. Based on our results, we were able to optimize conditions to maximize glycerol LC with acceptable EE.

As expected, the AG and ACG beads reflected the FTIR absorbance peaks from their parent molecules i.e. alginate, glycerol and chitosan (Figure 6). The spectral position of peaks identified in the AG and ACG polymer treatments were relatively similar with slight shifts suggesting changes in fundamental structure between the two polymers. The ACG treatment revealed narrower and more intense bands at regions that reflect new hydrogen bonds between chitosan and alginate and stretching of -COO groups reflecting polyelectrolyte complex formations. The -COO signal is more intense in the ACG beads compared to the AG beads (Figure 6). Greater intensity in the ACG suggest stronger ionic bonds between chitosan amine groups and carboxyl groups of alginate. In the ACG beads, peak bands shifted to a lower energy with an increase in the strength of the ionic interactions. The evidence of the interaction between alginate and chitosan can be seen in the broadening of some bands and increased intensity of others.

Alginate is a common encapsulating polymer used to protect bioactive nutrients from gastrointestinal environment and promote delivery to selective locations within the gut [25,26]. Alginate has a pKa of 3.5 and is relatively insoluble in acidic pH making it an attractive biopolymer for minimizing nutrient release in the gastric region of the gut [27]. Furthermore, the calcium-alginate combination has been shown to dominate the bead structure and form “egg-box” junctions acting as cationic bridges that impart stability and prevent penetration of the layer [18,28]. However, alginate ionic cross-linkages may have reduced stability as a result of greater solubility in the presence of monovalent cations [29]. The combination of a cationic polymer like chitosan interacts with alginate to modify thermal and functional properties and increase structure stability [18]. The slow release of glycerol in acidic buffer (pH 2) was as expected since alginate is insoluble at low pH. The greater response with the ACG beads is reflective of the increased stability of the biopolymers and presence of a stronger outer matrix. At higher pH there was almost complete release of glycerol. The alginate polymer resulted in more rapid release whereas the combination of alginate and chitosan showed a steady rate of glycerol release. These results are consistent with those reported earlier [13,17,23].

Fermentation data with mixed cultures of rumen microbes clearly show that both polymers provided some degree of protection to glycerol from microbial attack. Addition of free glycerol resulted in increased microbial fermentation as evidenced in the increased total SCFA concentration. The decrease in ruminal acetate and increase in propionate is a classic response to the availability of rapidly fermentable energy source. Encapsulating glycerol in alginate or alginate-chitosan resulted in minimal release of glycerol into the rumen culture and maximized glycerol protection from microbial fermentation. Similar protection from rumen microbes was reported for linseed oil encapsulated with alginate and alginate chitosan [30]. Alginate and chitosan biopolymers have been extensively used to protect delivery of pharmaceutical drugs in the gastrointestinal tract but limited use is reported in the ruminal environment.

Our data show that encapsulation of glycerol with alginate or alginate-chitosan can reduce the amount of free glycerol released in a pH 6 buffer and increase amount of glycerol delivered to the lower digestive tract.

Compared with alginate, the alginate-chitosan mix was more effective in increasing stability of the molecule in all intestinal environments. The LC and EE of glycerol was greater with the alginate-chitosan mix than alginate alone. *In vitro* fermentation with mixed cultures of rumen microbes further demonstrate the viability of the alginate-chitosan mix to provide protection for glycerol from microbial attack. *In vivo* experiments will need to be conducted to determine if indeed the combination of alginate-chitosan polymer can result in greater and steady amounts of glycerol delivery to the lower gut.

## Figures and Tables

**Table 1:** Effect of glycerol either unprotected (G) or encapsulated in alginate (AG), and alginate-chitosan (ACG) polymers on *in vitro* rumen microbial fermentation (n = 3)

Items	Hour	Treatment1)				SEM

		Con	G	AG	ACG	
Culture pH	0	6.24	6.22	6.19	6.14	0.037
	24	5.34	5.34	5.33	5.34	0.019
Short chain fatty acids (SCFA)						
Total (mM)	0	47.7	47.4	49.7	49.7	2.06
	24	135.5^a^	140.0^a^	112.7^b^	116.3^b^	1.65
Acetate (A, mol %)	0	61.3	57.5	58.2	58.1	1.64
	24	50.2^b^	41.9^c^	51.6^ab^	52.6^a^	0.68
Propionate (P, mol %)	0	22.2	22.1	21.5	21.3	1.64
	24	27.4^c^	32.6^a^	28.7^b^	29.3^b^	0.47
Butyrate (B, mol %)	0	9.0	8.9	9.4	8.5	0.61
	24	18.8^b^	20.4^a^	15.5^c^	13.9^d^	0.25
Valerate (V, mol %)	0	3.3	4.4	4.2	4.2	0.36
	24	1.9^b^	3.4^a^	2.1^b^	2.2^b^	0.14
BCSCFA, mol %	0	7.9	7.1	6.8	7.8	1.35
	24	1.6^ab^	1.5^b^	1.9^a^	1.8^a^	0.10
A:P	0	2.8	2.6	2.7	2.8	0.23
	24	1.8a	1.3^b^	1.8^a^	1.8^a^	0.05

SEM, standard error of means; BCSCFA, branched-chain SCFA.

1) Con, control (no glycerol); G, unprotected glycerol; AG, glycerol encapsulated in alginate; ACG, glycerol encapsulated in alginate-chitosan.

a–d Means within a row with different superscripts differ (p<0.05).
